# Distal transsylvian keyhole approach for unruptured anterior circulation small aneurysms

**DOI:** 10.1007/s00701-017-3378-7

**Published:** 2017-11-14

**Authors:** Ririko Takeda, Hiroki Kurita

**Affiliations:** Department of Cerebrovascular Surgery, International Medical Center, Saitama Medical University, 1397-1 Yamane, Hidaka City, Saitama 350-1298 Japan

**Keywords:** Keyhole approach, Transsylvian approach, Cerebral aneurysm, Clipping

## Abstract

**Background:**

To reduce complications associated with conventional pterional craniotomy, a transsylvian keyhole approach for unruptured small anterior circulation aneurysms is proposed.

**Methods:**

A 7-cm linear scalp incision is made along the hairline, beginning at the zygoma, followed by minimal temporal muscle dissection. Two burr holes are drilled out at McCarty’s point and the temporal bone, and a 3-cm equilateral triangle bone flap is made, whose apex is located above the sylvian point. After the sphenoid ridge is drilled off, aneurysms are exposed and clipped with conventional microsurgical instruments.

**Conclusions:**

This approach permits access to aneurysms via the transsylvian corridor with a smaller area of potential injury of superficial structures.

**Electronic supplementary material:**

The online version of this article (10.1007/s00701-017-3378-7) contains supplementary material, which is available to authorized users.

## Introduction

In line with recent advances in endovascular surgery, the need for less invasive direct aneurysm surgery has been increasing. Some types of keyhole approaches have been developed as alternatives to the conventional pterional craniotomy. However, most reported approaches are via supraorbital or subfrontal routes [1, 3, 7] and have drawbacks including a narrow surgical corridor, need for special microsurgical instruments, and damage to the superior orbital nerve [4]. In this study, the surgical techniques and important factors that enable usual pterional conventional microsurgical manipulations for anterior-circulation aneurysms via a keyhole approach are described.

## Relevant surgical anatomy

It is important to understand of the running of a facial nerve for its preservation in this approach. At the axial level of the upper edge of the zygomatic arch, the mean distance between the anterior border of the tragus and the most posterior branch of the facial nerve is 15.3 mm (range, 11.0–22.9 mm; SD, 3.5 mm) [6]. The branches of the facial nerve that innervates the orbicularis and frontalis muscle are located in the surface of superficial layers of deep temporal fascia and cross at a mean distance of 40.4 mm (range, 35.2–45.6 mm; SD, 3.3 mm) above the lateral canthus of the eye [6]. The most posterior temporal branch to the frontalis muscle that intersected the superior temporal line is located a mean distance of 34.9 mm (range, 29.1–40.6 mm; SD, 4.4 mm) posterosuperior to the lateral canthus of the eye [6].

## Description of the technique

### Craniotomy

**Fig. 1:**
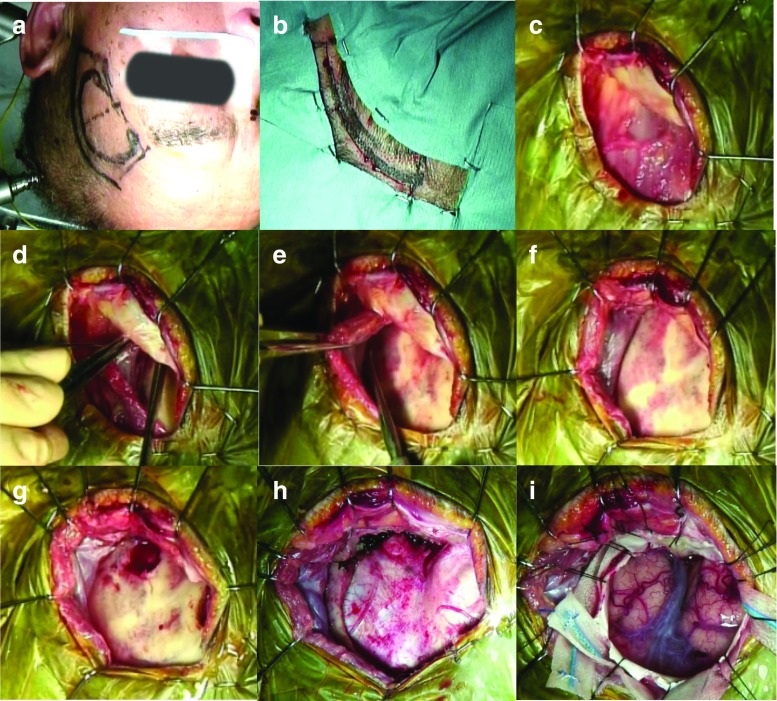
Craniotomy techniques. **a**, **b** Skin incision design. **c**-**i** Subfascial dissection without cutting the temporal muscle

After injection of lidocaine to the supraorbital and infraorbital nerves as preemptive analgesia, the skin incision is started at the level of the upper rim of the zygomatic arch and 2.0 cm anterior to the external acoustic meatus. The incision curves forward, passing 5 cm lateral from the lateral cantus of the eye, and ends at about 7 cm in length inside the hairline (Fig.1a, b). This design of the skin incision contains a sufficient safety margin to avoid facial nerve injury. The temporal fascia is sharply cut, and subfascial dissection is performed, protecting the temporal branch of the facial nerve [2]. After the fascia is reflected anteriorly along with the skin flap (Fig. 1c), the temporal muscle is dissected—beginning at McCarty’s point from the temporal plane—by the retrograde dissection method [5], and the bulky temporal muscle is retracted laterally so as not to disturb visualization along the sphenoid ridge (Fig. 1d–f). The dissection needs no incision of temporal muscle. Two burr holes are drilled at McCarty’s point and in temporal bone (Fig. 1g), and the bone flap is removed (Fig. 1h). The sphenoid ridge is removed as in the conventional pterional approach. The dura mater is cut in a semi-arched shape and opened to a size of 30–40 mm (Fig. 1i).

Because of minimal preparation and dissection of bones and muscles, iatrogenic surgical trauma, cranial deformities, and temporal muscle atrophy are significantly decreased postoperatively (Fig. 6).

We use a perforator to shape the two burr holes in this approach as well as conventional pterional craniotomy, because it is a familiar and routine procedure for us. However, to minimize the further bone lost and avoid the use of calcium phosphates, the use of a drill instead of a perforator is recommended.

### Microsurgical technique

**Fig. 2:**
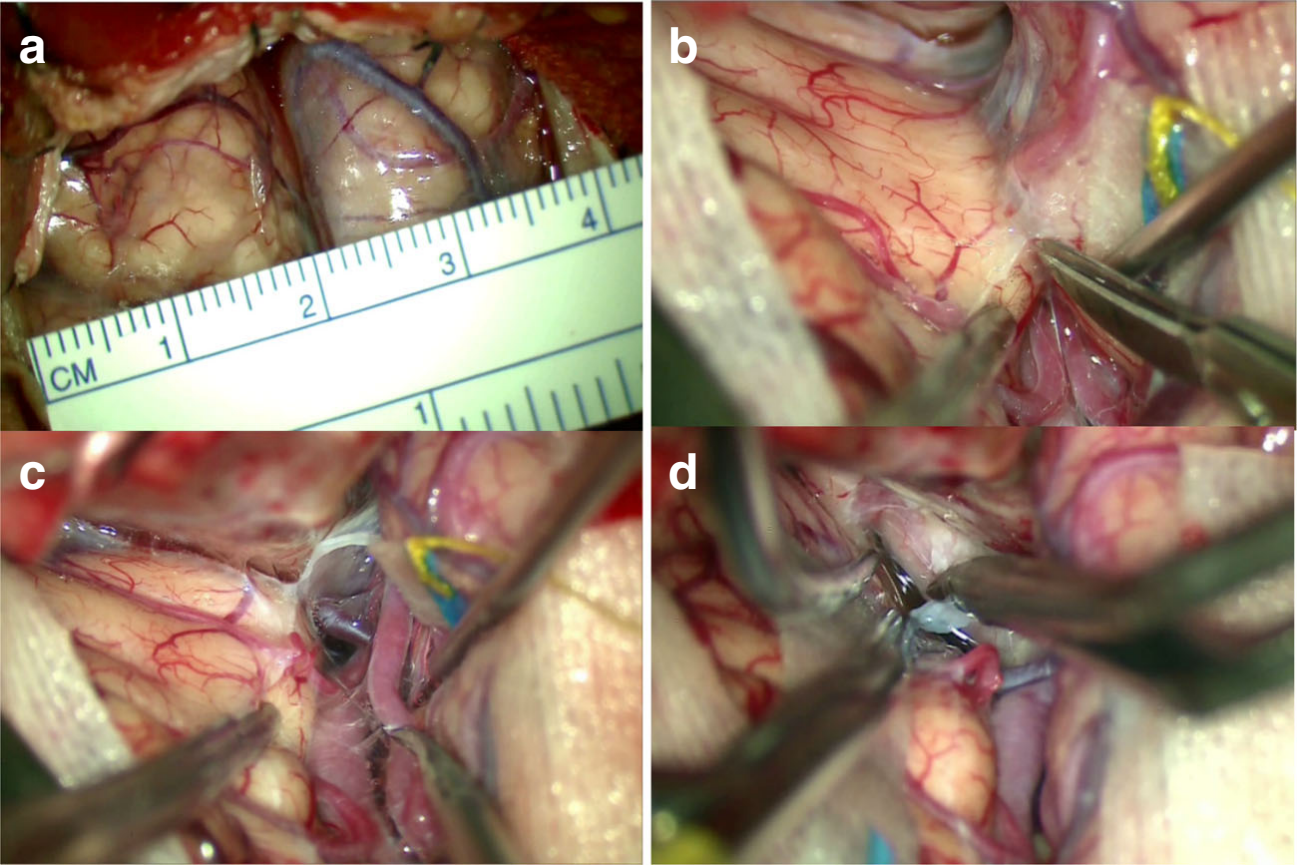
Microscopic views. **a** Dura opened area. **b**-**d** Sufficient working space for sharp dissection of the sylvian fissure

In the microsurgical view, we can see the sylvian fissure at the center and the frontal and temporal lobes on both sides. The sylvian point [8], which is the confluence of three rami of the sylvian fissure and a suitable to start for opening the sylvian fissure because of the wide subarachnoid space, is included in the operative view. The sphenoid ridge is removed to the lateral edge of the superior orbital fissure. Therefore, we can see that this size of craniotomy provides sufficient working space and a familiar view for sharp dissection of the sylvian fissure from the sylvian point to the internal carotid cistern, equal to a conventional large pterional craniotomy (Fig. 2a-d).

## Indications

**Fig. 3:**
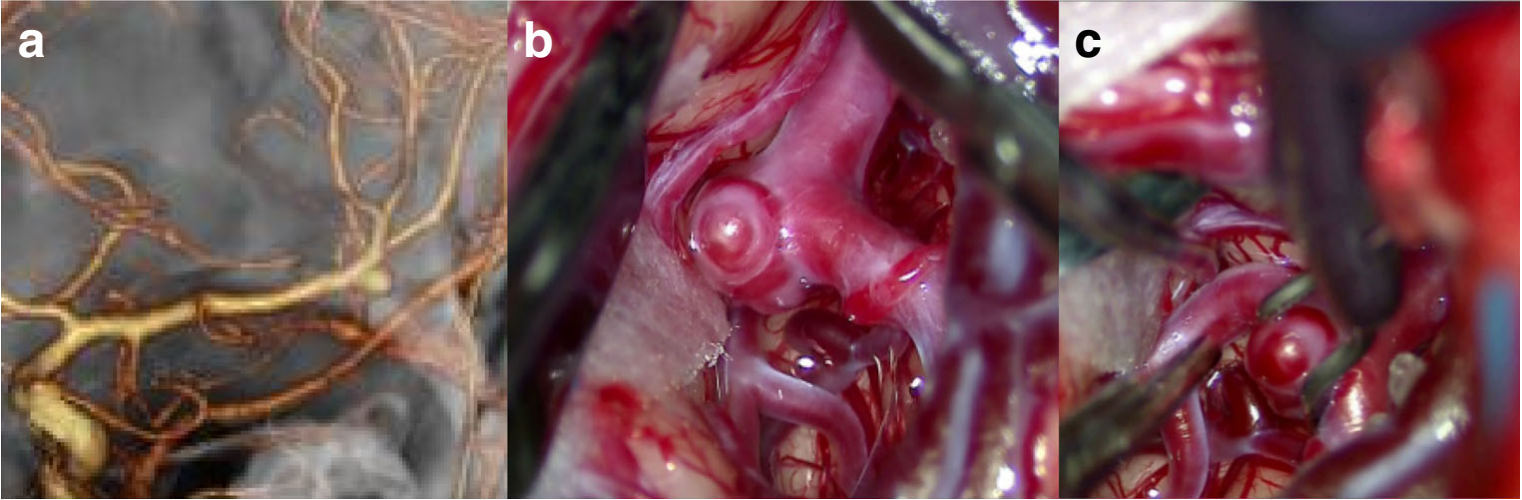
Representative case of a left MCA aneurysm. **a** Preoperative 3D–CT angiogram. **b**, **c** Intraoperative views

**Fig. 4:**
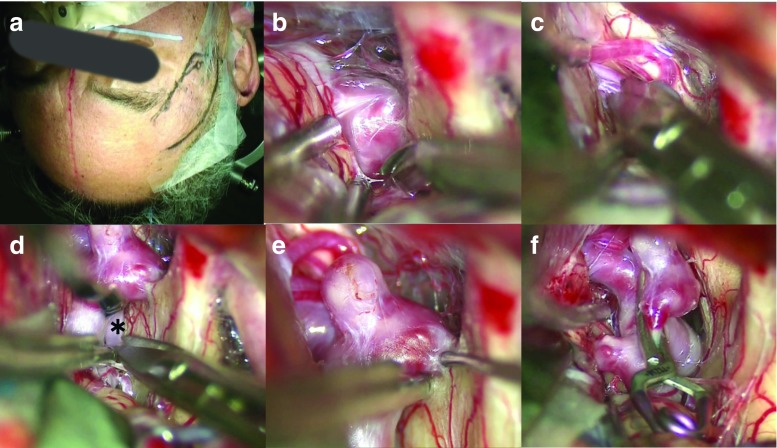
Representative case of an Acom aneurysm. **a** Skin incision. **b** Opening of the suprachiasmatic cistern, and **c** interhemispheric fissure. **d** Preserving left A1(*). **e** Dissection of the dome. **f** Clipping of the aneurysm

**Fig. 5:**
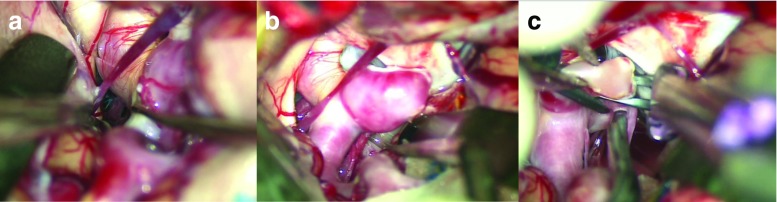
Representative case of a right Pcom aneurysm. Dissection **a** medial and **b** lateral of the ICA. **c** Clipping of the aneurysm

**Fig. 6:**
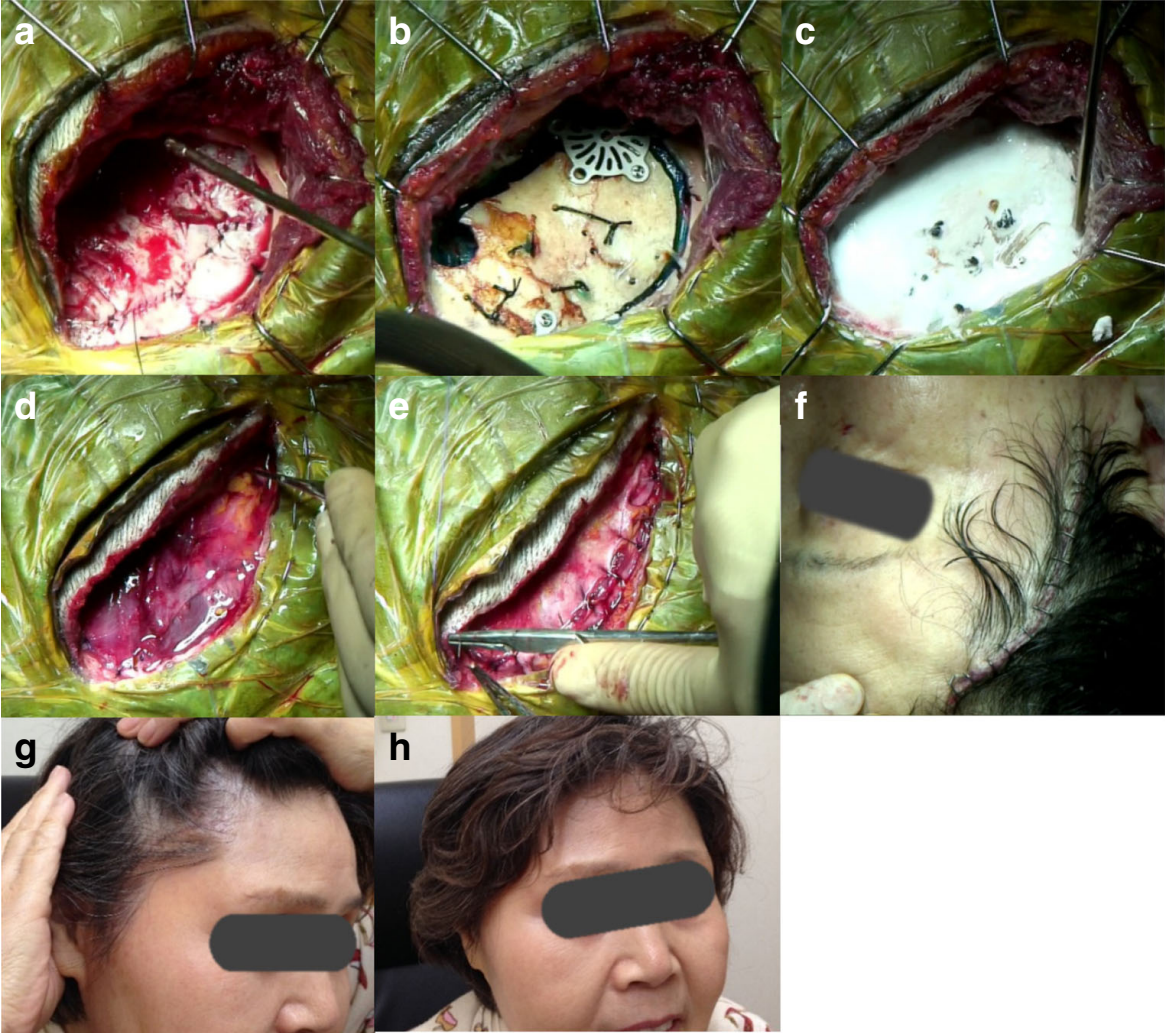
Closing of the craniotomy. **a**, **b** Bone flap design. **c** Calcium phosphate-filled bone defects. **d**, **e** Temporal muscle and fascia reconstituted to the original position. **f** Photograph after the operation. **g**, **h** Photographs 1 month after surgery

Good candidates for this approach are selected patients with small unruptured aneurysms in the anterior circulation, which include: (1) all middle cerebral artery (MCA) bifurcation aneurysms (Fig. 3); (2) laterally and posteriorly projected internal carotid-posterior communication (IC-PC) aneurysms; (3) internal carotid-anterior choroidal (IC-AchoA) artery aneurysms (Fig. 5); (4) anteriorly projected anterior communicating (Acom) aneurysms (Fig. 4). For posteriorly projected IC-PC aneurysms, additional dissection of retrocarotid cisterns and temporal lobe retraction are necessary (anterior temporal approach). Anteriorly projected Acom aneurysms are also safely clipped with additional exposure and dissection of ipsilateral suprachiasmatic cisterns and the interhemispheric fissure. However, some superiorly or posteriorly projected Acom aneurysms require wide opening of the interhemispheric fissure, so that the conventional pterional approach is better for these aneurysms (Figs. 5 and 6).

Conversely, this technique is contraindicated for patients with ruptured aneurysms, complex aneurysms including large or partially thrombosed aneurysms, and those requiring removal of the anterior clinoid process or bypass.

## Limitations

This approach provides the same clear anatomic exposure as the conventional pterional approach, and it provides a familiar view to neurosurgeons. The sylvian fissure is seen in the center of the surgical window. The approaches from the sylvian fissure to the carotid cisterns, suprachiasmatic cistern, and interhemispheric cistern are the same as with the conventional pterional approach, and the viewing angle along with the microscopic lighting is also equal to wide craniotomy.

The surgical procedure is carried out with conventional microsurgical, bayonet-shaped short instruments, without specialized skills. No particular instruments and devices—for example, a neuroendoscope to compensate for the limitation of the visualization—are needed.

More severe brain traction is not needed in this approach. As in the conventional pterional approach, wide opening of the fissure and cisterns requires less brain traction in dissection of the aneurysm.

However, the small size of the bone flap allows a more limited angle of clip applier insertion.

## How to avoid complications

The key point to overcoming the problem of the limited angle of clip applier insertion is complete dissection of the aneurysm complex from surrounding structures, which makes the aneurysms ‘movable’ to permit changing their direction to that most suitable for neck clipping.

## Specific perioperative considerations

Certainly, this approach cannot be applied to all patients with aneurysms. With careful evaluation of each patient for the possibility of proximal control, direction of the aneurysm, and application of the aneurysm clip, this approach should constitute an effective craniotomy technique.

## Specific information to give to the patient about surgery and potential risks

This approach has a smaller area than the conventional pterional approach, but results in a pleasing cosmetic outcome while minimizing the likelihood of procedure-related morbidity.

## Electronic supplementary material

ESM 1 The video illustrates the necessary procedures to the pterional keyhole approach for unruptured aneurysmal clipping, showing a left MCA aneurysm as an example. (WMV 114835 kb)

## References

[1] Cheng WY, Lee HT, Sun MH, Shen CC (2006). A pterion keyhole approach for the treatment of anterior circulation aneurysms. Minim Invasive Neurosurg.

[2] Coscarella E, Vishteh G, Spetzler RF, Seoane E, Zabramski JM (2000). Subfascial and submascular methods of temporal muscle dissection and their relationship to the frontal branch of the facial nerve. J Neurosurg.

[3] Mori K, Yamamoto T, Oyama K, Watanabe M, Nonaka S, Hara T, Honma K (2008). Lateral supraorbital keyhole approach to clip unruptured anterior communicating artery aneurysm. Minim Invasive Neurosurg.

[4] Nathal E, Gomez-Amador JL (2005). Anatomic and surgical basis of the sphenoid ridge keyhole approach for cerebral aneurysms. Neurosurgery.

[5] Oikawa S, Mizuno M, Muraoka S, Kobayashi S (1996). Retrograde dissection of the temporalis muscle preventing muscle atrophy for pterional craniotomy. Technical note. J Neurosurg.

[6] Poblete T, Jiang X, Komune N, Matsushima K, Rhoton AL (2015). Preservation of the facial nerves to the frontalis muscle during pterional craniotomy. J Neurosurg.

[7] Reisch R, Perneczky A, Filippi R (2003). Surgical technique of the supraorbital key-hole craniotomy. Surg Neurol.

[8] Ture U, Yasagil DC, Al-Mefty O, Yasagil MG (1999). Topographic anatomy of the insular region. J Neurosurg.

